# Helicobacter pylori infection is not related to increased carotid intima-media thickness in general population

**DOI:** 10.1038/s41598-018-32465-4

**Published:** 2018-09-21

**Authors:** Yunfei Feng, Weibin Zhou, Luo Luo, Weiwei Xu

**Affiliations:** 1 Department of Endocrinology, the First Affiliated Hospital, College of Medicine, Zhejiang University, #79, Qingchun Road, Hangzhou, Zhejiang, 310003 China; 2 Information Center, the First Affiliated Hospital, College of Medicine, Zhejiang University, #79, Qingchun Road, Hangzhou, Zhejiang, 310003 China

## Abstract

The aim is to determine whether there is an independent association between Hp infection and carotid intima-media thickness (CIMT) in a cross-section observational study. Among of 14588 routine health check-up participants, 13770 subjects underwent the ^13^C-urea breath test (^13^C-UBT) and ultrasound measurement of CIMT. Traditional atherosclerotic risk factors were also recorded. The ratio of increased CIMT in Hp positive group (28.6%) was not significant difference compared with Hp negative group (29.7%) (p = 0.164). The HP infection rates was no significant difference between increased CIMT (38.4%) and non- increased CIMT (39.7%) patients. However, all the traditional atherosclerotic risk factors including age, gender, BMI, waistline, total cholesterol, low density lipoprotein cholesterol, very low density lipoprotein cholesterol, high density lipoprotein cholesterol, triglycerides, free fatty acid, homocysteine, systolic and diastolic blood pressure, fasting plasma glucose and C reactive protein were different between increased CIMT and non- increased CIMT participants. The odds of Hp infection for CIMT risk (OR 0.948; 95% CI 0.879–1.022; P = 0.164) was not higher in binary logistic regression analysis even after adjustment for traditional risk factors (OR 1.118; 95% CI 0.958–1.306; P = 0.157). Our study found no evidence of association between CIMT and HP infection.

## Introduction

Coronary atherosclerosis is a process of disease involving multiple factors, in which chronic inflammation plays a key role, while other hazards such as dyslipidemia also are attributed to the nosogenesis of atherosclerosis^1^. Established cardiovascular risk hazard factors, such as elevated blood pressure, dyslipidemia, inflammation and immune factors, endothelial dysfunction and plaque disruption, can not interpret all cases and therefore new risk factors are widely researched^2^. Inflammation play a crucial part in the development of atherosclerosis and has drawn more and more attention. Multiple infections, including Helicobacter pylori (Hp) infection, can enhance the production of proinflammatory cytokines, which may be a pivotal hazard factor for atherosclerosis^3^. It is also suggested that infectious factors may play a key part in the pathogenesis of atherosclerosis with continuous low-level inflammation of the vessels leading to endothelial dysfunction^1^. However, from the epidemiological point of some studies, the impact of Hp on the pathogenesis of coronary heart disease (CHD) is still controversial^4^, and previously published studies of infection and cardiovascular disease generally tend to be biased and lack a sufficient sample capacity^5^. Moreover, the mechanisms of Hp infection leading to coronary atherosclerosis, and the association between Hp infection and clinical and laboratory hazard factors including blood pressure, smoking, blood glucose and lipids is not completely studied.

Hp which belongings to a gram-negative bacterium, microaerophilic and spirally shaped, settles on the gastric mucosa of about 50% of all adults^6^. Hp infection and its evoked chronic inflammation can not only lead to gastric diseases, such as chronic gastritis, digestive ulcers, and gastric malignancies^6^, but also extragastric diseases including atherosclerosis^3^. More and more evidences show that carotid intima-media thickness (CIMT) measured by ultrasonography is an incipient sign of atherosclerosis and can be applied as an alternative index of clinical and subclinical atherosclerosis^7^. In this cross-sectional investigation, we attempted to study the relationship between Hp infection with the increased carotid inter-media thickness.

## Results

Among the enrolled 14588 general participants, 13770 individuals have both Hp test and CIMT measurements. According to ^13^C-UBT, 5418 participants (39.3%) were Hp positive and 8352 participants (60.7%) were Hp negative. Participants’s characteristics are shown at Tables 1 and 2. The increased CIMT ratio in Hp positive group (28.6%) was not statistically significant in comparison with Hp negative group (29.7%) (p = 0.164). However, there were statistical differences of age, gender, BMI, LDL-C, HDL-C, FFA, DBP and FPG between Hp positive and negative group. There was no significant difference between the HP infection rates of increased CIMT (38.4%) and non- increased CIMT (39.7%) participants in the study. The traditional atherosclerotic risk factors including age, gender, BMI, waistline, TC, LDL-C, VLDL-C, TG, FFA, Hcy, SBP, DBP, FPG and CRP of the increased CIMT subjects were higher than those of the non- increased CIMT subjects while it is the opposite of HDL-C. The odds of Hp infection for CIMT risk (OR 0.948; 95% CI 0.879–1.022; P = 0.164) was not higher in binary logistic regression analysis even after adjustment for age, gender, BMI, waistline, TC, LDL-C, VLDL-C, HDL-C, TG, FFA, Hcy, SBP, DBP, FPG and CRP (OR 1.118; 95% CI 0.958–1.306; P = 0.157).

**Table 1:** Subject characteristics in Hp positive and negative groups (n = 13770).

Features	Hp (+) group (n = 5418)	Hp (−) group (n = 8352)	p value
Age (years)	52.0 ± 12.1	52.8 ± 12.8	<0.001
Men (n)	3387 (62.5%)	4890 (58.5%)	<0.001
BMI, kg/m^2^	24.3 ± 6.4	23.9 ± 3.3	<0.001
Waistline, cm	86.8 ± 9.8	86.4 ± 9.2	0.086
TC, mmol/L	4.80 ± 0.90	4.79 ± 0.91	0.465
LDL-C, mmol/L	2.78 ± 0.75	2.74 ± 0.75	<0.01
VLDL-C, mmol/L	0.70 ± 0.42	0.70 ± 0.48	0.940
HDL-C, mmol/L	1.32 ± 0.35	1.35 ± 0.36	<0.001
TG, mmol/L	1.53 ± 1.12	1.54 ± 1.22	0.849
FFA, mmol/L	0.37 ± 0.16	0.38 ± 0.16	<0.05
Hcy, umol/L	13.4 ± 6.7	13.1 ± 6.4	0.063
SBP, mmHg	128.6 ± 18.5	128.3 ± 21.2	0.448
DBP, mmHg	78.0 ± 11.6	77.4 ± 11.3	<0.01
FPG, mmol/L	5.20 ± 1.22	5.14 ± 1.05	<0.01
CRP, mg/L	2.09 ± 7.33	1.78 ± 3.57	0.110
ICIMT (n)	1549 (28.6%)	2480 (29.7%)	0.164

Hp, Helicobacter pylori; BMI, body mass index; TC, total cholesterol; LDL-C, low density lipoprotein cholesterol; VLDL-C, very low density lipoprotein cholesterol; HDL-C, high density lipoprotein cholesterol; TG, triglycerides; FFA, free fatty acid; Hcy, homocysteine; SBP, systolic blood pressure; DBP, diastolic blood pressure; FPG, fasting plasma glucose; ICIMT, increased carotid intima-media thickness.

**Table 2:** Subject characteristics in increased and non- increased carotid intima-media thickness groups (n = 13770).

Features	ICIMT group (n = 4029)	NICIMT group (n = 9741)	p value
Age (years)	65.1 ± 11.2	47.3 ± 8.8	<0.001
Men (n)	2841 (70.5%)	5436 (55.8%)	<0.001
BMI, kg/m^2^	24.4 ± 3.0	23.9 ± 5.3	<0.001
Waistline, cm	88.5 ± 9.3	86.0 ± 9.5	<0.001
TC, mmol/L	4.83 ± 0.98	4.78 ± 0.87	<0.01
LDL-C, mmol/L	2.80 ± 0.82	2.74 ± 0.72	<0.001
VLDL-C, mmol/L	0.71 ± 0.45	0.70 ± 0.46	<0.05
HDL-C, mmol/L	1.32 ± 0.36	1.34 ± 0.35	<0.05
TG, mmol/L	1.57 ± 1.12	1.52 ± 1.20	<0.05
FFA, mmol/L	0.39 ± 0.17	0.38 ± 0.16	<0.01
Hcy, umol/L	14.3 ± 6.6	12.9 ± 6.5	<0.001
SBP, mmHg	137.5 ± 18.6	124.7 ± 19.6	<0.001
DBP, mmHg	80.0 ± 11.1	76.7 ± 11.4	<0.001
FPG, mmol/L	5.52 ± 1.41	5.02 ± 0.94	<0.001
CRP, mg/L	2.57 ± 8.34	1.75 ± 4.51	<0.01
Hp (n)	1549 (38.4%)	3869 (39.7%)	0.164

Hp, Helicobacter pylori; BMI, body mass index; TC, total cholesterol; LDL-C, low density lipoprotein cholesterol; VLDL-C, very low density lipoprotein cholesterol; HDL-C, high density lipoprotein cholesterol; TG, triglycerides; FFA, free fatty acid; Hcy, homocysteine; SBP, systolic blood pressure; DBP, diastolic blood pressure; FPG, fasting plasma glucose; ICIMT, increased carotid intima-media thickness; NICIMT, no increased carotid intima-media thickness.

## Discussion

It is necessary to search new risk factors since traditional atherosclerotic risk factors may explain only 50% of its etiology^8^. Although accumulating data of many cross-sectional investigation indicate that many infections such as Hp may be responsible for the development of atherosclerosis^3,9^, these studies were hampered by their small size and the consequent bias of selection^10^, so there is a dispute of this conclusion^11^. And they failed to take full account of the possible insufficient adjustment for confounder variables. There is sizeable proof implying that ultrasound measurements of early atherosclerosis are clinically significant. The measurement of CIMT is one of the alternative indicators of atherosclerosis. Several studies have indicated the relation between intima-media thickness (IMT) and cardiovascular and cerebrovascular events^7^. Though Akbas *et al*.^12^ discovered that Hp positivity was associated with increased CIMT, it is controversial that whether there is a relationship between Hp and CIMT^13^. In this survey, we investigated the relationship between Hp infection and CIMT in general subjects. Though numerous more recent publications concerning these associations are available^14,15^, our results were consistent with previous cohort studies that indicate no significant correlation between Hp infection and the average CIMT^13^.

Although Epidemiologic studies on basis of serological results have implied that there is a relationship between atherosclerosis and chronic Hp infection^9^, Wald *et al*.^16^ found Hp seropositivity was not associated with ischemic heart disease in a routine medical examination of 21520 professional men. Atherosclerosis Risk in Communities Study Investigators concluded that both the traditional hazard factors and the average carotid IMT have no correlation with the HP seropositive^17^. These differences among ethics, age factors and the study protocol (qualitative and quantitative analysis) might interpret, at least partially, such conflicts. As a result, the positive relation of reports in smaller studies may be caused by accidental or published bias^9^ (or both). Oppositely a large number of prospective studies showed there was no obvious relationship between Hp and cardiovascular disease^18,19^.

Many identified risk factors may affect the progress of cardiovascular disease. Lots of studies have discovered that Hp infection is more related to age, male gender and social status, which are associated with CHD^20,21^. Thus, it is imperative to take into account the underlying confounding factors. HDL-C plays a pivotal function in the inverse cholesterol transport, protecting LDL from oxidation and reducing lipoprotein related peroxides. The most crucial role of HDL is to accelerate cholesterol outflow from the cells. HDL-C also possesses antioxidant and anti-inflammatory activities. The high plasma levels of HDL-C have many proven roles to prevent the progress of atherosclerosis. Low concentration of HDL-C is recognized to be a proverbial hazard factor for coronary heart illness^22^. Our study found that HDL-C, was lower in the increased CIMT subjects and Hp positive group compared with non- increased CIMT and Hp negative groups, further supporting the hypothesis of confounding factors. Another population based study in china demonstrated that HP infection was related to declined serum levels of HDL, but not associated with the ponderance of coronary atherosclerosis^22^, which is consistent with our results.

CRP, as an innate immunity pattern-recognition molecule or a sensitive inflammatory marker, can activate endothelial cells to express adhesion molecule, induce monocytes to release cytokine, and stimulate the complement cascade, which directly lead to the inflammatory state of atherosclerosis and has been revealed to play an important part in the pathophysiology of plaque development/progression in CHD subjects^23^. Furthermore, several researches have suggested that CRP is an independent and novel risk assessment atherosclerotic marker for CHD. Therefore, high CRP levels may identify subjects capable of producing a significant inflammatory response to pathogens and other stress factors. This capacity has a complex genetic control and was recently shown to enhance the risk of atherosclerosis^23^. However, the present study found higher CRP level of increased CIMT was not associated with Hp infection.

Our study had a large population of Chinese individuals and the present study does not support a role of Hp in association with CIMT. There are limitations deserve considerations in this investigation. First, the present research is only a cross-sectional study instead of a prospective, case-control study, so the outcomes fail to offer information about the causality between Hp infection and coronary atherosclerosis. Secondly, there is a gap between ^13^C-UBT and the gold standard tests for Hp infection and the CIMT was not quantified. Thirdly, our research samples only include Chinese. The findings may not be applicable to other ethnic groups. Fourthly, in our study, though drugs including histamine 2 receptor antagonists, antibiotics, colloidal bismuth subcitrate or proton pump inhibitors, which could affect the results of 13 C urea breath tests (13C-UBT), were not permitted to use in the patients within a 1-month period before screening. However, these therapy before 1 month will affect sensitivity and specificity of breath test and increase the percentage of false negative results. Just as our study took part in East China near Shanghai, and the living style is developed as Western developed countries. In recent studies, the H. pylori prevalence in China was found to be significantly lower than that reported in previous studies. The overall H. pylori prevalence in urban China was found to be 31.9%^24^ and 43.8%^25^. These studies are consistent with our study. Nevertheless, this survey does not oppose that the greater inflammatory virulence of the Hp strain may increase the risk of CHD^26^, since strains of Hp expressing the virulent cytotoxin-associated gene product A (CagA) has stronger correlation with CHD than other strains by stimulating more of the immune system^27^. The bacterium could trigger the acute onset of ischemic heart disease by stimulating platelet aggregation^28^. However, some studies^29^ found no association between CagA positivity and atherosclerosis. The state of CagA is not identified, so it is not clear whether the CagA positive strain is related to the ponderance of coronary atherosclerosis. We suggest that future researches of HP strains in large prospective trials and intervention studies are required to ascertain the possible function of HP in atherosclerosis.

## Methods

### Subjects

In this cross-sectional study, 14588 general participants were recruited consecutively from the Department of Health Care Center, the First Affiliated Hospital, College of Medicine, Zhejiang University during a routine health check-up from January 1, 2015 to September 30, 2016. The subjects who had a history of hypertension, hyperlipidemia, diabetes, stroke, and cardiovascular disease were excluded in this investigation. In addition, drugs including histamine 2 receptor antagonists, antibiotics, colloidal bismuth subcitrate or proton pump inhibitors, which could affect the results of ^13^C urea breath tests (^13^C-UBT), were not permitted to use in the patients within a 1-month period before screening. Among these participants, 13770 individuals have both Hp test and CIMT measurements and have conformed to the above conditions.

This study was carried out in accordance with the Helsinki Declaration and approved by the Ethics Committee of the First Affiliated Hospital, College of Medicine, Zhejiang University. All subjects gave written informed consent before the study.

### ^13^C-urea breath test

Hp infection was diagnosed on the basis of the result of fasting ^13^C-UBT. Briefly the ^13^C-UBT was carried out according to the manufacturer’s instructions: subjects underwent an 8-h fast, and a baseline breath specimen was gathered. After 10 min, subjects drank 100 mL of water dissolved with 75 mg of ^13^C isotope-labeled urea (Beijing Boran Pharmaceutical Co. Ltd., China) and a second respiration sample was taken 30 min later; the infrared heterodyne ratiometry (Beijing Huaheng Anbang Company, China) were used to analyzed the respiration samples. According to the ^13^C-UBT Hp infection results, participants were divided into the Hp positive group and Hp negative group.

### Anthropometric measurements

The height measurement was accurate to 0.1 centimeters, and the weight was accurate to 0.1 kilograms. The body mass index (BMI) was obtained by calculation of the weight (kg)/the square of height (m^2^). The waist circumference accurate to mm was surveyed at the middle level between the lowest rib edge and the iliac crest. Systolic blood pressure (SBP) and diastolic blood pressure (DBP) were surveyedd on the right arm and three measured values of each individual were written down, and the average value was recorded as as the final result.

### Risk factors assessment

Levels of traditional atherosclerotic risk factors including total cholesterol (TC), low density lipoprotein cholesterol (LDL-C), very low density lipoprotein cholesterol (VLDL-C), high density lipoprotein cholesterol (HDL-C), triglycerides (TG), free fatty acid (FFA), homocysteine (Hcy), fasting plasma glucose (FPG) and C reactive protein (CRP) were also determined from the same sample.

### Measurement of carotid intima-media thickness

The CIMT was performed by a disciplined ultrasonographer using a Philips Ultrasound System HD11XE (Royal Dutch Philips Electronics Ltd., Amsterdam, Netherlands) by means of a 10-MHz linear probe. The subjects were checked in the supine position with the neck rotated away from imaging transducer. The definition of CIMT was refer to the distance between the blood-intima interface and the media-adventitia interface. All procedures were conducted on both sides of two longitudinal images of each common carotid artery. CIMT was checked at the 10 mm distal end of the common carotid artery. Three CIMT measurements were acquired from each side to calculate the average value of CIMT. If the CIMT is equal or more than 1 mm, we consider the carotid intima- media is increased^30^.

### Statistical analysis

Data was analyzed with a statistical software package IBM-SPSS Statistics (International Business Machines Corp., Armonk, New York) version 23. The total numbers (proportions) of categorical variables and the mean ± standard deviation (SD) of continuous variables were used to identify the subjects. Normality of the continuous variables was examined by the Kolmogorov–Smirnov test. For the comparison between two groups, the Student’s t-test was used for continuous variables and the chi-square test for categorical variables. To analyze the relationship between Hp infection with CIMT, Binary logistic regression test was employed by adjusting age, gender, blood pressure, cholesterol, and other cardiovascular risk factors. These primary analyses adopted 95% CIs and two-tailed P values. Differences were considered statistically significant when p < 0.05.
